# In memory of the well-renowned Chinese cellular biologist Shien Sher-Pu

**DOI:** 10.1093/procel/pwac002

**Published:** 2022-07-15

**Authors:** Nan Shen, Shen Liu

**Affiliations:** School of Foreign Languages, Anhui Agricultural University, Hefei 230022, China; School of Humanities and Social Sciences, Anhui Agricultural University, Hefei 230022, China; Research Center for Philosophy of Physics, University of Science and Technology of China, Hefei 230022, China

**Figure 1.**
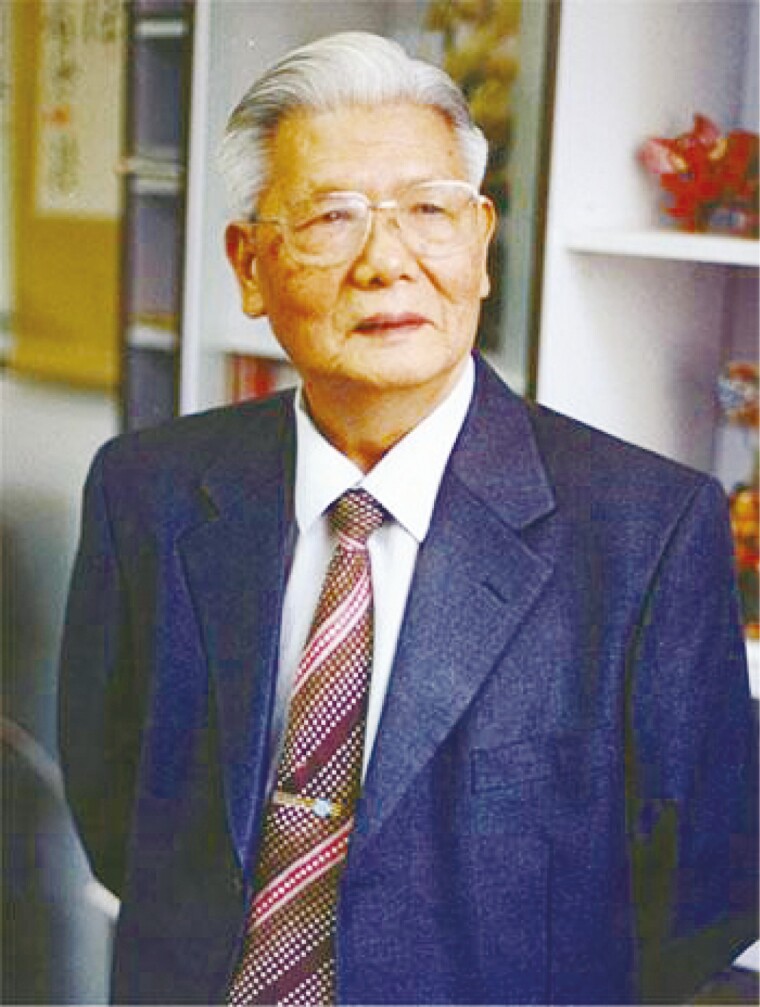
Academician Shien Sher-Pu.

**Figure 2.**
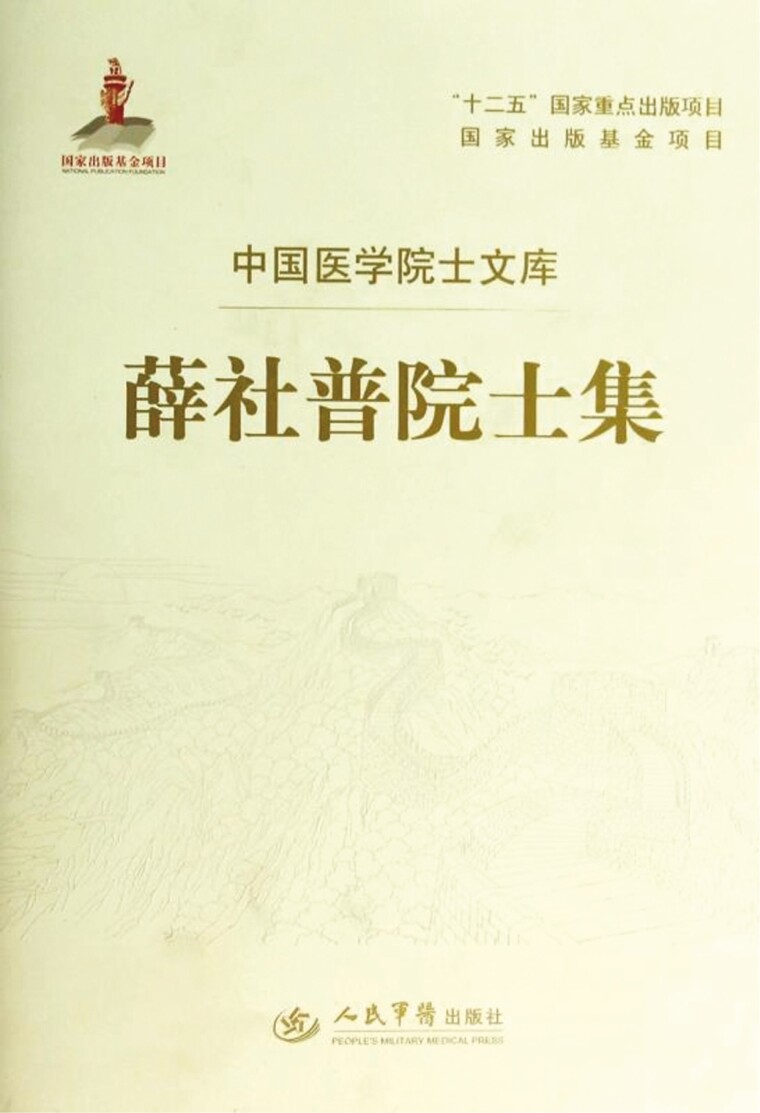
Collected works of academicians Shien Sher-Pu.

Academician Shien Sher-Pu (薛社普), one of the pioneers of cytobiology and reproductive biology research in China, initiated a research regarding red blood cells (RBC) nucleation mechanism in cell growth and differentiation regulation. He had achieved breakthrough achievements in the development of male birth control pill and made an important contribution to the research of reproductive biology and cytopharmacology (Figs. 1 and 2).

In 1917, Shien Sher-Pu was born in a rural family in Zhuwan Village, Gujing Town, Xinhui County, Guangdong Province. His father worked as a Chinese laborer in North America; therefore, he could not constantly stay at home. Thus, the whole family lived on the hardworking of his mother, Mrs. Zhao. Shien Sher-Pu’s mother lived a harsh life by doing farm work and taking care of three children at home. Unfortunately, such overwork led to her early death at the age of 39. Shien was 8 years old at that time and was adopted by his aunt before starting primary school at the age of 9. Nevertheless, Shien Sher-Pu lived a happy life although his childhood was materially difficult. The beautiful mountains he often climbed with his friends, the lush trees, and lovely birds and animals in his hometown all furnished his strong love and curiosity of nature, which was sustained in his memory for a long time and ended up becoming the comfort of his entire life.

Shien Sher-Pu had been very introverted since childhood, but he was very diligent, which attributed to his excellent grades at primary school. After graduating from primary school in 1932, Shien Sher-Pu was admitted to the famous Guangya Middle School in Guangdong Province. Guangya Middle School, evolving out of Kwang Ya Academy founded by Zhang Zhi-dong who had been a famous official in the late Qing Dynasty, was the best middle school in Guangdong Province with a beautiful natural environment, excellent teachers, and advanced facilities, and the school only accepted the most exceptional students. The 6 years of life in Guangya Middle School exerted a profound influence on Shien Sher-Pu’s life and laid the foundation for his academic style of attaching equal emphasis to science and humanities. Afterwards, he often spoke of what Guangya Middle School meant to his life.

In 1938, Shien Sher-Pu graduated from senior high school and attended National Central University (NCU). National Central University, located in Nanjing, was the predecessor of current Nanjing University. It was one of the most outstanding institutions of higher education in China at that time. It had been moved to Chongqing due to the war and Shien Sher-Pu studied in the department of natural history. In order to save enough living expense, he had been working as a part-time biology teacher in a nearby middle school since freshman year. Shien Sher-Pu often took students to collect animal and plant specimens in nature, and he also learned excellent drawing skills which later proved very useful in his research. After graduating from university, due to his academic excellence, Shien Sher-Pu was offered a job as an assistant to Professor Wang Xi-cheng, head of the department. Wang’s tutor in Germany was Professor Hans Spemann, winner of the 1935 Nobel Prize in Physiology or Medicine and an internationally renowned embryologist. Deeply influenced by his tutor, Wang Xi-cheng established a series of courses in embryology immediately after returning to National Central University and became the founder of embryonic research in China. During the period as a teaching assistant, Shien Sher-Pu had passionate sense of duty and demonstrated strong curiosity for science and excellent experimental ability. He gradually developed a strong interest in experimental embryology and was determined to take it as his lifelong career.

In 1943, Shien got married and had a daughter next year. However, he did not just settle down to live a simple life, but actively strived for the opportunity of further study on a higher platform. In 1946, National Central University was moved back to Nanjing. Shien Sher-Pu passed the examination of state-financed students’ studying abroad and was admitted to the Department of Zoology at the University of Chicago. In December 1947, Shien finally arrived there to study under the guidance of famous neurobiologist Professor Paul Weiss. Learning of Shien’s interest in experimental embryology, Paul Weiss, a generous person, recommended him to Washington University in St. Louis in 1948 to study for a doctorate with Professor Viktor Hamburger.

Professor Hamburger, a strict and affable Germans scholar, owned a distinguished reputation in the international field of cell differentiation. At that time, he had also taught two outstanding students who would later become internationally famous: Stanley Cohen from America and Rita Levi-Montalcini from Italy. Under Hamburger’s guidance, they became the earliest to identify and purify bioactive cytokine, namely nerve growth factor and epidermal growth factor, for which they won the Nobel Prize in 1986. There is no doubt that such a high-level international academic environment had exerted profound influence on Shien Sher-Pu. With Professor Hamburger’s careful guidance, Shien began to carry out experiments to examine the development and differentiation of nervous system by using chick embryo. In a difficult experiment of nerve tissue transplantation, Shien accurately transplanted the cervical spinal cord to the thoracic level under anatomic lens, and found that the cells in the cervical spinal motor region that were supposed to degenerate and die could survive in the thoracic environment and differentiate into a new kind of Terni pregang lionic type. That great and innovative result showed that the growth, degeneration, and differentiation of nerve cells are closely related to their microenvironment and can be regulated under certain conditions (Shieh, 1951).

After New China was founded, Shien Sher-Pu had a yearning to return and serve his motherland. In the spring of 1951, Shien declined the invitation from foreign experts to stay and resolutely returned to China with the help of his tutor. After returning home, Shien devoted himself to the nation’s education and scientific research. He was successively engaged in teaching and scientific research in Dalian Medical College, Harbin Medical College, Beijing Normal University and Peking Union Medical College, etc. In Chinese Academy of Medical Sciences, Prof. Shien established China’s first laboratory to study chicken embryos and autoradiography in the Institute of Medicine. During that period, Professor Shien had been diligently working in his research and continuously made a number of world-leading achievements, among which two works are of great significance. One is the research intended to use chicken embryos as experimental environment to study the effect of normally developing embryos on the differentiation of tumor cells. Shien Sher-Pu found that chicken embryo germ cells could change their differentiation type under certain conditions or become cancerous after virus infection. In addition, malignant tumor cells have the potential to be induced to differentiate into embryonic tissue in the embryo (Shieh and Pu, 1951; 1957). Second, Shien used the advanced isotopic autoradiography technique to prove that yolk sphere of chicken embryo could not absorb the methionine labeled with the isotope ^35^S, so they had no function of protein anabolism and self-renewal, and could not form cells themselves (Shieh and Pu, 1958). That finding strongly refuted the view propounded by the former Soviet scientists that the yolk sphere of chicken embryo could evolve into endoderm cell and blood islands.

In 1970, Shien Sher-Pu started to study Male Birth Control by gossypol at Institute of Experimental Medicine, Chinese Academy of Medical Sciences. Since the 1950s, booming population had been directly impacting the economy and social stability through the world, which made population control a major social issue to be resolved. In this case, if the effective male contraception medicine could be developed, it would definitely benefit China and other countries in terms of controlling population. Shien Sher-Pu, experienced in the field of embryonic development, kept in touch with many domestic and international institutes to study male contraception by using rattus, mus, guinea pigs, and macaca mulatta as animal models, which lasted for more than 20 years. During that time, Shien also led the team to study the generation and differentiation regulation of germ cell and the mechanism of relevant drugs. They conducted the systematical study of China’s antisperm birth control drugs like gossypol and *tripterygium wilfordii*, and established the animal experimental research model, effect evaluation index and a set of quantitative detection techniques (Shieh et al., 1983). Meanwhile, Shien Sher-Pu was funded by WHO and the United States Population Council, and was invited to carry out academic exchanges and scientific research cooperation over male contraception in nine cities in America.

Shien Sher-Pu once briefed his scientific work as “One idea, two cells.” “One idea” could be explained as the investigation into cell differentiation, and “two cells” as spermatoblast and erythrocyte. In 1980s, Shien began to study the erythroid denucleation mechanism, so as to prevent tumor by inducing the differentiation of tumor cells, which elevated his scientific career to a new level. Shien once said: “The K562 cell is a human erythroleukemia cell, and the mouse MEL cell is also a erythroleukemia cell. Both cells will lose their hemoglobin expression after turning into tumor. Instead of being condensed and discarded, the cell nucleus will multiply and divide out of control, forming deadly cancer cells.” Therefore, Shien assumed that the EDDF (erythroid differentiation/denucleation factor) emerged during the phase of erythrocyte terminal differentiation, and the erythroleukemia cell may fail to perform terminal differentiation for the lack of that factor. Therefore, if the EDDF from normal erythrocyte was injected into the erythroleukemia cell, it could be induced into differentiation and reverse the malignancy of the tumor. Based on the assumption, Shien Sher-Pu accomplished the innovative experiment with much trial and great efforts. It was proved that the existence of EDDF in reticulocytes could enhance terminal differentiation of erythrocyte.

Aside from all his accomplishments, Shien Sher-Pu also led the team to purify EDDF protein from the reticulocyte of mus, rattus, and rabbits, and tested its amino acid sequence and cDNA fragment sequence. Six members of the EDDF gene family cloned during different stages of terminal differentiation were proved to be newly found gene sequence. Later, Dr. Weiss probed into the functions of EDDF gene cloned by Shien Sher-Pu, and found that it could stabilize the α-hemoglobin and was related to β-thalassemia, which further proved that the series of EDDF genes cloned by Shien played an important role in the terminal differentiation and functional regulation of erythrocyte.

On 10 March 2017, Professor Shien Sher-Pu passed away at the age of 100 at Peking Union Medical College Hospital. Professor Shien Sher-Pu devoted his whole life to the frontier of scientific research and contributed to laying a foundation for higher education in new China. Advocating the team spirit in scientific research, Professor Shien Sher-Pu paid much attention to the young talents and cultivated many outstanding students. Most of them contributed to scientific research and teaching in related fields by inheriting the legacy of their teacher Shien Sher-Pu.

Professor Shien Sher-Pu’s academic life is not only splendid, but also thought-provoking. He had been conducting fearless and pioneering exploration among the vast biological world and daring to question authorities in order to raise his own independent opinions. After returning to China, Professor Shien was capable of attaining a good balance between individual pursuits and national interests, and achieved great accomplishments in various fields. That manifested his astounding academic diversities and deep-rooted patriotism. Professor Shien Sher-Pu’s life is undoubtedly the guiding model of Chinese intellectuals, and his noble character and spirit of persistence should be understood and learned by all scientific researchers.
